# Influence of primary students’ self-regulated learning profiles on their rating of a technology-enhanced learning environment for mathematics

**DOI:** 10.3389/fpsyg.2023.1074371

**Published:** 2023-04-17

**Authors:** David Bednorz, Svenja Bruhn

**Affiliations:** ^1^Department of Mathematics Education, IPN—Leibniz Institute for Science and Mathematics Education, Kiel, Germany; ^2^Faculty of Mathematics, University of Duisburg-Essen, Essen, Germany

**Keywords:** technology-enhanced learning environments, metacognition, motivation, self-regulation, primary school, digital mathematics education

## Abstract

The importance of learning technologies for mathematics education is increasing as new opportunities arise for mathematics education for all students, in school and at home. These so-called *technology-enhanced learning environments* (TELEs) incorporating technology with mathematical content are useful for developing mathematical knowledge and can simultaneously foster self-regulated learning (SRL) and motivational learning in mathematics. However, how do primary students’ differences in their SRL and motivation affect their rating of the quality of mathematical TELEs? To answer this research question, we asked third and fourth-grade primary students (*n* = 115) to evaluate both their SRL, including metacognition and motivation, and the quality characteristics of the ANTON application, a frequently and intensively used TELE in Germany. Using a person-centered research approach by conducting a cluster analysis, we identified three SRL profiles of primary students—*motivated self-learners, non-motivated self-learners,* and *average motivated non-self-learners*—who differ in their ratings of the quality characteristics of the TELE (output variables). Our results highlight that motivated self-learners and non-motivated self-learners vary significantly in their rating of the *adequacy* of the TELE to their mathematical learning and highly but not significantly concerning the TELE’s *reward* system. Moreover, differences existed between the motivated self-learners and the average motivated non-self-learners regarding their rating of the characteristic *differentiation*. Based on these findings, we assume that technical elements associated with adequacy, differentiation, and rewards of mathematical TELEs should be tailorable to the needs of individuals and groups of primary schoolchildren.

## 1. Introduction

By conceptualizing the increasing digitalization of the mathematical learning processes, the umbrella term *technology-enhanced learning environment* (TELE) is frequently used in mathematics education research to “cover all those circumstances where technology plays a significant role in making learning more effective, efficient or enjoyable” (Goodyear and Retalis, 2011, p. 8). While the attribute “effective” alludes to aspects of students’ self-regulated learning (SRL), the attribute “enjoyable” clearly refers to the significance of affective components of thriving digital mathematical learning. Hence, it is reasonable that mathematics education researchers increasingly emphasize the importance of students’ self-regulatory and motivational skills in digital mathematical learning processes (e.g., Schrunk et al., 2014; Panadero, 2017). However, most research studies explore the effects of TELEs on students’ mathematical learning by designing, implementing, and evaluating digital learning (e.g., Dettori and Persico, 2011; Higgins et al., 2017). Consequently, mathematics education research examining how students’ SRL abilities and motivation influence their use of mathematical TELEs and how students’ diverse learning requirements can or cannot be met by digital technologies is lacking. Prospectively, detailed insights into this allow mathematics educators to meaningfully include TELEs in their daily teaching. Moreover, most digital technologies frequently used in primary schools worldwide are developed by profit-orientated companies and not codesigned by mathematics education researchers (Braun et al., 2000; KMK, 2021). Thus, it is expedient to scientifically examine these existing TELEs concerning their potential for students’ self-regulated learning processes. Therefore, this paper aims to investigate how primary schoolchildren’s self-perceived self-regulatory abilities affect their rating of the mathematical quality of an exemplary German TELE.

## 2. Theoretical background

Learning processes regulated by students are considered an essential component of successful mathematics education (Schunk and Greene, 2017; Trias et al., 2021). As Steffens (2008) and Krauthausen (2018) stated, due to the numerous technical opportunities in designing digital mathematical learning environments, TELEs are expected to be particularly suited to facilitating self-regulated and motivated learning for primary students. Thus, we subsequently present relevant aspects of the characteristics of digital mathematical technologies (Section 2.1) and students’ SRL in TELEs (Section 2.2) to identify the research gap for our empirical study (Section 2.3).

### 2.1. Characteristics of TELEs in primary mathematics education

Media are fundamental elements of mathematical teaching and learning processes, and the advanced technological developments of various mathematical hardware and software have expanded the educational possibilities for mathematics learning in the early school years (Kyza et al., 2009; Goodyear and Retalis, 2011; Goodwin and Highfield, 2013; Moyer-Packenham et al., 2015). Therefore, the term TELE is used to adequately cover all the characteristics of digital technologies that lead to meaningful, competence-orientated, and sustainable mathematical learning for all primary students (Kurvinen et al., 2020).

Striving to systematize the tremendous variety of digital technologies in mathematics education, Sinclaire (2016) identified three groups: mobile personal devices (e.g., calculators, mobile phones, tablets, and computers), tangible designs for mathematical experience (e.g., 3D printers, motion visualizers, touch technologies, and virtual reality), and social technologies (e.g., social media and networking). For primary mathematics education, the TIMSS (2020) report found that fourth-grade students increasingly use mobile personal devices such as iPads, as they are easy to use, cost-effective, and have diverse application possibilities. In their long-term observational study with first to third-grade students, Levinsen and Sørensen (2018) found that “the iPad, with its potential for providing agency, constitutes a strong factor for students’ motivation, creativity, and relational learning” (p. 575) in mathematics. Moreover, iPads enable primary schoolchildren with special educational needs to access mathematics (Beal and Rosenblum, 2018).

Following the introduction of iPads for mathematical learning, a huge variety of mathematical applications were designed and programmed (e.g., Klinger and Walter, 2022), representing TELEs with varying levels of complexity. Since Trouche et al. (2012) found that, after about 2 decades of research, only a few digital technologies met mathematics education standards, the characteristics of TELEs for primary mathematics education need to be characterized in depth. For example, Walter (2018) highlights that mathematical TELEs are characterized by diverse mathematical potentials, such as synchronicity and integration of representation levels, structuring aids, fitting between action and mental operation, multitouch handling, and reduction of cognitive load. However, a key element of applications is that they have specific properties allowing only certain numbers and types of actions to be performed, encouraging students to show specific mathematical thinking processes (Webb, 2005; Herring et al., 2016). Here, three major characteristics of TELEs can be named that influence students’ mathematical learning: (1) Larkin and Milford (2018) determined that mathematical applications for primary schoolchildren (ages 5–11) often foster declarative rather than the conceptual, relational, and exploratory learning of mathematics promoted as core competencies in standards for mathematics education (e.g., OECD, 2020). (2) Due to technical or capability-related restrictions in programming mathematical applications, limited opportunities can be found for differentiated instructions (Tomlinson, 2005; Subban, 2006). Social differentiation (e.g., choosing single, partner, or group work), quantitative differentiation (e.g., varying the number of subtasks, selecting tasks, or adapting the processing pace), differentiation by media (e.g., presenting tasks both in written and auditory form), or various types of qualitative differentiation (e.g., additional explanations, tips, adjusting the difficulty of the tasks, and changing the representation level) may be implemented to meet the diverse needs of schoolchildren (Krauthausen, 2018). (3) Moreover, TELEs may involve various types of feedback, such as knowledge of performance, knowledge of results/responses, knowledge of correct responses, answer-until-correct feedback, multiple-try feedback, and especially elaborated feedback (Narciss, 2008) to foster students’ mathematical learning. These types of feedback create the basis for new, state-of-the-art, mathematical TELEs characterizing complex, intelligent tutoring systems (ITSs) and hence can respond adaptively to students’ input and support them in their mathematical learning processes (Kulik and Fletcher, 2016).

### 2.2. Self-regulatory and metacognitive mathematics learning with TELEs

Summarizing the previously presented characteristics of mathematical TELEs realized as applications with diverse technical restrictions (Section 2.1), using digital media in mathematics education cannot, *per se*, help students realize and manage their individual learning needs and guarantee sustainable mathematical learning processes (Walter and Dexel, 2020). However, Bernacki et al. (2011) highlight in their literature review that TELEs that sensibly include meaningful elements such as adequate feedback, qualitative differentiation options, or clear, addressee-related instruction can successfully increase students’ SRL skills (Section 2.2.1) as well as their motivation to learn mathematics (Section 2.2.2).

#### 2.2.1. Potential for using TELEs for students’ SRL

Generally, the term SRL “serves as a comprehensive framework for understanding how students become active agents of their learning process” (Fadlelmula et al., 2014, p. 1,355); therefore, primary students must monitor and, if necessary, adapt their learning by structuring the learning task, performing suitable strategies, or evaluating their solution (Bishop et al., 2020). This is true for performing mathematics in physical and technology-enhanced learning environments that especially enable students’ relational thinking (Carter et al., 2020).

In the discussion about a model description of SRL, a distinction is made between process models (e.g., Zimmerman, 1986, 2000; Zimmerman and Moylan, 2009) and component models (e.g., Boekaerts and Niemivirta, 2000; Boekaerts, 2011) that attribute situation-specific variables to students’ self-regulated actions differently (for an overview, see Panadero, 2017). Process models such as that of Zimmerman and Moylan (2009) consider three periodic phases—*forethought, performance or volitional control,* and *self-reflection*—and assume that SRL is context-specific and therefore applied differently by learners in different learning tasks. Focusing especially on digital learning tasks suitable for self-regulated learning processes, Bartolomé and Steffens (2011) presented three criteria of TELEs strongly related to stages of process-orientated SRL models: (1) “Learners should be encouraged to plan their learning activities”; (2) “Learners should receive appropriate feedback so they can monitor their learning”; and (3) “Learners should be given criteria so they can evaluate their own learning outcomes” (p. 23–24). Thus, special emphasis is placed on two key characteristics of TELEs (Section 2.1): Implementing different types of feedback, such as knowledge of performance, knowledge of correct responses, or elaborated feedback (Narciss, 2008), supports students’ self-regulatory learning processes in TELEs. Additionally, differentiation options (Krauthausen, 2018) of TELEs play a significant part in students’ planning and monitoring of their learning.

Since component SRL models focus intensively on the interrelationships between cognitive, metacognitive, motivational, and emotional components of SRL (Zeidner et al., 2000), they are well-suited to describing students’ learning processes in TELEs, as educational researchers tend to examine the design, computational, cognitive, social/cultural, and epistemological aspects of digital technologies (Balacheff et al., 2009). Strong emphasis is placed on students’ self-regulated learning strategies that have recently been differentiated for digital mathematics learning (e.g., Lai and Hwang, 2016; Greene et al., 2018): While cognitive SRL strategies focus mainly on repetition, elaboration, and organization of students’ mathematical learning processes (Pintrich and Garcia, 1994), metacognitive strategies focus on students’ planning, monitoring, and regulation skills (Zimmerman, 2000). Regarding metacognition as a core component of SRL, students need to know the *how* (procedural knowledge)*, when* (conditional knowledge), *and why* (declarative knowledge) to use mathematical procedures/strategies suitable for solving mathematical tasks in every learning environment (Winne and Azevedo, 2014). Focusing on SRL strategies, Nieto-Márquez et al.’s (2020) study on third to fifth-grade students working on digital mathematical games reveals the effects of this type of TELE on the self-regulation of students’ learning behavior. Specifically, they found that students’ abilities in applying cognitive and metacognitive SRL strategies and overriding habitual replies increased and were facilitated by the students’ motivational and inhibitory characteristics. This result strongly emphasizes the relationship not only between SRL and the use of digital mathematical technologies but also between the use of mathematical TELEs and students’ motivation to learn mathematics.

#### 2.2.2. Potential for using TELEs for students’ motivation

Strongly intertwined with SRL, motivation is relevant to students’ mathematical learning processes, as it influences the duration of learning, individual task-dependent choices, methods of learning, and successful learning (Schrunk et al., 2014). Regarding motivation as a multidimensional construct (Vansteenkiste et al., 2009), Star et al. (2014) define four highly discussed psychological aspects of student motivation that Abu and Kribushi (2022) extend by formulating relevant questions: (1) Individual *self-efficacy* concerns the question, “Can I do the task?”; (2) *Implicit theories of ability* that are either fixed or incremental in students are addressed by the question, “Can I improve my ability?”; (3) *Value beliefs* include aspects such as interest, utility, attainment, or cost of studying mathematics and are framed by the question, “Do I want to invest in and continue studying various subjects in mathematics?”; (4) The *learning climate* concerns social, psychological, and educational aspects of the (digital) learning environment and asks the question, “Does the curriculum allow me an opportunity for meaningful learning?”

Recent mathematics education studies highlight that digital mathematics technology positively affects students’ motivation (Chao et al., 2016), mathematical problem-solving, and attitude toward mathematics (Higgins et al., 2017). For instance, a randomized experimental study by Faber et al. (2017) emphasizes the impact of TELEs in the forms of *digital formative assessment tools* (DFATs), such as the Dutch *Snappet*, on third-grade students’ mathematical achievements and their motivation to learn mathematics. By focusing especially on intrinsic motivation as a predictor for mathematical achievement, divergent research results can be found. Pekrun et al. (2002) emphasized that students’ intrinsic motivation positively impacts emotional aspects such as hope, enjoyment, or pride but negatively impacts aspects such as boredom or hopelessness in learning mathematics. Contrarily, in their study with first to fourth-grade students, Garon-Carrier et al. (2016) established that intrinsic motivation does not naturally lead to mathematical achievement during elementary school but found that high-achieving students showed more intrinsic motivation for learning mathematics. However, how students’ intrinsic motivation influences their mathematics learning in TELEs remains minimally researched.

Moreover, a major advantage of TELEs is that they enable the implementation of *digital game-based learning* (DGBL) elements (Vankúš, 2021) with or without a *value-added design* (Mayer, 2019). This distinction refers to the application of specific game design characteristics (gamification) that encourage students’ conceptual and relational understanding of mathematics. Qian and Clark (2016) found that using DGBL as a student-centered approach to learning mathematics can be characterized as an enjoyable and interactive learning environment and that this appears to strongly impact students’ motivation (e.g., Zhao et al., 2021; Fadda et al., 2022). For instance, the broad review by Hussein et al. (2022) indicated that design-based learning might “improve academic performance, motivation, and attitudes towards learning” (p. 2860) of primary to secondary students.

### 2.3. Research questions

Summarizing the theoretical aspects, TELEs in the form of applications, on iPads or similar personal mobile devices, with specific characteristics, such as *adequate* mathematical complexity and task design, sufficient *instruction, differentiation* options with various feedback types, and DGBL (*reward*) elements, have the potential to facilitate substantial relational mathematics learning for primary students (Section 2.1). Therefore, recent studies evidence that using digital technologies in mathematics education develops students’ SRL strategies (Section 2.2.1) and, especially, increases their motivation to learn and achieve in mathematics (Section 2.2.2). However, it remains unclear how students’ individual SRL abilities in learning mathematics affect their mathematics learning in TELEs with specific characteristics. Therefore, this paper examines how primary students differ in their SRL abilities and how these differences affect their ratings in frequently used and yet minimally researched German TELEs. Therefore, we expect to gain insight into the requirements TELEs must fulfill to meet students’ diverse needs based on their SRL abilities. Thus, we aim to answer the following research questions:

1. What student profiles can be identified based on their self-estimated SRL abilities and motivation?
2. Do student SRL profiles differ in their ratings of the quality characteristics of a TELE?

## 3. Materials and methods

In this study, which used empirical methods to gather the data, we focused on the exemplary German TELE ANTON (Section 3.1). We asked third and fourth-grade students (Section 3.2) to evaluate the quality characteristics of this mathematical learning environment as well as their self-regulatory, metacognitive, and motivational skills. For this purpose, two test instruments were developed and sensitively applied to the mathematical and textual abilities of primary school students (Section 3.3). By using a person-centered research approach, we aimed to analyze the quantitative data (Section 3.4) and identify student profiles to answer the two research questions.

### 3.1. The exemplary TELE: ANTON

In Germany, mathematical TELEs for primary mathematics education take the form of applications (Klinger and Walter, 2022). Since the beginning of the COVID-19 pandemic, the free TELE, the ANTON learning platform for schools, has been widely used in primary schools in Germany. In its imprint, the developer characterized ANTON as “a universal learning platform (web & mobile) for schools and students that can be used for independent self-learning as well as for interactive learning in a classroom context” (Solocode GmbH, 2021). Schoolchildren from the first to the tenth grade can use the ANTON TELE for self-regulated learning and practicing mathematics (as well as other subjects such as German language, English, arts, and natural and social science) in curriculum-orientated and grade-specific exercises. The instructions for the various mathematical exercises are represented in short sentences both auditorily and visually to enable students’ SRL with ANTON. Although the scope and difficulty of the exercises cannot be adjusted by the students or the teacher, ANTON offers differentiation tips that students can use independently to solve the exercise problems. As a reward system for gamification and motivation, students receive coins for completed exercises that they can use to play small non-mathematical arcade games or customize their avatars with new items.

Cofinanced by the European Regional Development Fund (ERDF) of the EU, ANTON was developed in 2017 and is continuously updated by the German Solocode GmbH, involving a wide variety of employees, such as software developers, artists, educators, and a few researchers. Although this TELE, like many others, has not been adequately evaluated by mathematics education researchers, it has received strong positive feedback from users, such as parents, students, and mathematics educators. For instance, ANTON is partly purchased *via* statewide school licenses for entire states (e.g., Lower Saxony). Following this, ANTON is expected to meet important professional standards for digital mathematics education with an emphasis on students’ self-regulatory strategies and metacognitive and motivational skills. Therefore, it appears particularly significant to examine its characteristics more closely.

### 3.2. Sample

A total of 115 third and fourth graders with an average age of 9.8 years (*MIN* = 8.6 *years*; *MAX* = 11.6 *years*) from five different German primary schools participated in this study voluntarily and with parental consent. The sample comprised 62.6% of the third-grade students in the selected schools; 51.3% of the sample were girls, and 48.7% were boys. At the time of the data collection, June 8^th^ to 29^th^, 2021, the mathematics teacher for all the third and fourth-grade classes had practiced with ANTON with their students for several months at different intensities. A total of 56 students from two primary schools used ANTON obligatorily in homework or distance learning and had their results checked by the mathematics educators. However, they did not practice mathematics with ANTON in the classroom. At another school, 11 third and fourth-grade students used ANTON only in distance learning but not in mathematics classrooms and voluntarily only for homework. Contrarily, 18 students from one primary school worked only in school on various mathematical content but did not use ANTON in homework or distance learning. A total of 30 third and fourth graders from the last primary school worked with ANTON obligatorily both in mathematics classrooms and for homework or distance learning. Hence, all participating students were individually familiar with practicing various mathematical topics with this specific TELE.

The data collection was conducted by four students from Bielefeld University and one of the two authors of this paper. A test administration manual was created, describing concrete instructions for the test procedure to ensure comparable test results for the participating third and fourth-grade students. For example, it was determined that the students were first tested with the test instrument “ANTON, Mathematics, and Me” followed by the second test instrument, “Mathematics, School, and Me,” within 4 days, excluding the weekend. The tests were anonymized by assigning individual codes to identify the school, gender, grade, and any special educational needs of the students and allow the association of the two test instruments with each student to enable comparative analysis of the results.

### 3.3. Test instruments

Two test instruments were created to capture two constructs, (1) self-regulatory, metacognitive, and motivational skills and (2) the mathematical quality characteristics of ANTON, to answer the research question on how primary students evaluate ANTON and differ in their SRL and motivation.

The first test instrument, “Mathematics, School, and Me,” assessed the SRL strategies, metacognition, and motivation of the participating third and fourth graders, the most common subscales for assessing self-regulated learning abilities. By developing this test instrument, we adapted existing instruments from PALMA (project for the analysis of performance development in mathematics; Vom Hofe et al., 2002; Pekrun et al., 2006) and PISA (Programme for International Student Assessment; Artelt et al., 2001). We adapted the test instrument by rephrasing the SRL strategies for primary mathematical activities and focusing on the students’ mathematical abilities and experiences. Thus, we shortened the item scope and adjusted the items linguistically. The redesign resulted in 15 items—five item blocks with three items each (exemplary items in Table 1; full item list in supplementary material). In completing the test instruments, the students evaluated three categories of SRL strategies—repetition strategies (SRL1), elaboration strategies (SRL2), and controlling strategies (SRL3). Students also evaluated their motivation to learn mathematics (MO) and metacognitive strategies to learn mathematics (MC) again using a 4-point Likert scale classically represented by tables with four labeled tick boxes.

**Table 1:** Examples of the self-regulated learning test instrument items.

Construct	Item	Item wording
Repetition (SRL)	SRL1.1	I first look at every single math problem very carefully so that I can then work on it.
Elaboration (SRL)	SRL2.3	When I study math, I understand new content better if it ties into old topics.
Control (SRL)	SRL3.2	Before I start working on a math problem, I try to remember or write down everything important.
Motivation	MO.2	I also enjoy doing math in my free time and solving math problems.
Metacognition	MC.3	I first think very carefully about how to work on the math problem and write down a plan.

Regarding the quality characteristics of the digital learning test instrument “ANTON, Mathematics, and Me,” the students were asked to evaluate the mathematical quality of ANTON. The self-designed instrument focused on 11 items with questions on the characteristics of mathematical TELEs (Section 2.1). These included productive and relational mathematics practice adequately matching students’ mathematical (A1.1, A1.2, A4, and A7) and technical (A2) abilities, clear instructions (A3.1 and A3.2), opportunities for differentiation (A5.1 and A5.2), and motivation through a reward system as a gamification aspect (e.g., A6.1 and A6.2). The exemplary items are shown in Table 2 (full item list in supplementary material). While these items were measured using a 4-point Likert scale and were child-friendly (e.g., represented by four different smileys, thumb movements, or a slider on a continuum), four items required an open response from students (A1.2, A3.2, A5.2, and A6.2). Due to third and fourth-grade students’ limited experience with test instruments, the additional open items were designed to obtain a more accurate rating from the children than the Likert scale rating.

**Table 2:** Examples of the quality characteristics of digital learning test instrument items.

Construct	Item	Item wording
Adequate practicing	A1.1 A1.2	How do you like practicing math with ANTON? Tick and explain.
Instructions	A3.1 A3.2	How helpful do you find the explanations of the different math problems? Tick and explain.
Differentiation	A5.1 A5.2	ANTON gives you tips for some of the exercises. How helpful do you find the tips? Tick and explain.
Reward system	A6.1 A6.2	What do you think about collecting coins in the math exercises and then playing games for them? Tick and explain.

### 3.4. Data analysis methods

The collected data from both test instruments were first edited by transferring the students’ answers to the items on the four-point Likert scale to corresponding numerical values from 1 to 4. Responses marked between two scale values were described by the arithmetic mean (e.g., a cross marked between 2 and 3 was defined as 2.5) to help the third and fourth graders answer the questions according to the test instructions.

To analyze the additional open items of the quality characteristics of the TELE in the test instrument, we first transcribed students’ answers by considering the exact wording as well as the spelling and grammatical errors. Using a qualitative content analysis (Mayring, 2015), all answers were inductively categorized and systematized into a category system for each of the four items. Subsequently, we matched the various qualitative categories to the four numerical values in the 4-point Likert scale. By calculating the arithmetic mean of the closed and open item parts, the items A1, A3, A5, and A6 were represented by numerical values. Moreover, the sum of each item block and the mean were determined to obtain the results for the subscales of the self-regulated learning test instrument.

We then analyzed the descriptive quantitative data, such as reliability statistics, average expression, and correlation of the items within and between the two test instruments. For these analyses, we used the *R package psych* (*Vers. 2.2.5*; Revelle, 2022). To answer RQ1 on the students’ SRL profiles, we z-standardized the data and performed a cluster analysis with the most important SRL strategies (evaluation, control, and repetition; Section 2.2.1), metacognition and motivation. Thus, we first used a hierarchical clustering procedure followed by identifying artificial cases and deleting them from the dataset using the nearest neighbor method and a dendrogram. By subsequently applying the Ward method, using the elbow criterion and the dendrogram, we determined the number of clusters that best fitted the data. The result was a three-cluster solution determined through a K-means cluster analysis. To answer RQ2 on the influence of the SRL profiles on the students’ rating on the TELE, we conducted a comparative analysis of the mean *z*-scores for the different quality characteristics (adequacy, instructions, differentiation, and rewards) for each SRL profile using the Kruskal–Wallis test.

## 4. Results

To legitimize the ensuing in-depth quantitative analyses, we first calculated the reliability of the aggregated subscales of both test instruments. The three subscales for the self-regulated learning test instrument comprised three items each. The Cronbach’s alpha values of the three SRL strategies—repetition (SRL1), elaboration (SRL2), and control (SRL3)—were 0.57, 0.65, and 0.45, respectively. Due to the low reliability of the control, we have not used this variable for further analyses. The reliability for the metacognition (MC) and motivation (MO) items was represented by Cronbach’s alpha values of 0.68 and 0.51, respectively. To achieve a better reliability score for the motivation, we excluded the first item (M.1) and obtained a Spearman–Brown coefficient of 0.64. For the quality characteristics test instrument, the adequacy subscale comprised five items (A1.1, A1.2, A2, A4, and A7), and the Cronbach’s alpha was 0.67. As the subscales instruction (A3.1 and A3.2), differentiation (A5.1 and A5.2), and rewards (A6.1 and A6.) comprised two items each, we calculated the Spearman–Brown coefficient (Eisinga et al., 2013). The analysis results for instruction, differentiation, and reward item coefficients were 0.62, 0.81, and 0.73, respectively.

Based on the reduced number of variables, we conducted a cluster analysis as well as a comparative analysis to answer both research questions—the question focusing on analyzing student profiles based on their self-perceived self-regulatory strategies, metacognitive, and motivational skills (RQ 1, Section 4.2) and the question differentiating these profiles based on their ratings of the characteristics of ANTON (RQ 2, Section 4.3).

### 4.1. Three primary students’ SRL profiles

**Figure 1:**
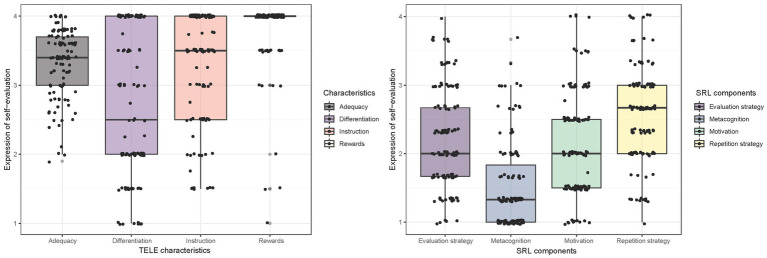
Boxplots of the rating of the quality characteristics of the technology-enhanced learning environment (TELE) and self-estimated self-regulated learning (SRL) components.

Figure 1 shows box plots for expressing the students’ self-perceived SRL, metacognition, and motivation (right box plot) as well as the primary students’ ratings of the quality characteristics of the TELE (left box plot). Initially focusing only on the four remaining SRL components, these show means close to the midpoint of the four-point Likert scale (i.e., 2). While, compared with the other components, the SRL strategies reach the highest expressions with *M* = 2.61 for the repetition strategy and *M* = 2.22 for the evaluation strategy, students’ motivation is moderately strong with *M* = 2.18. Moreover, the metacognition reaches the lowest expression with *M* = 1.54. Additionally, Table 3 shows the correlation within and between the two test instruments. For the SRL test instrument only, the largest correlation was obtained for motivation and the evaluation strategy, *r* = 0.48 (*p* < 0.01), corresponding to a moderately strong positive correlation. Furthermore, the analysis revealed that the two SRL strategies, repetition and evaluation, are positively correlated, *r* = 0.43 (*p* < 0.01). Furthermore, the repetition strategy and motivation were also significantly correlated, *r* = 0.41 (*p* < 0.01). Moreover, motivation and metacognition were significantly correlated, *r* = 0.26 (*p* < 0.01), as were metacognition and the SRL evaluation strategy, *r* = 0.35 (*p* < 0.01).

**Table 3:** Means, standard deviations, and correlations with confidence intervals.

Variable	*M*	*SD*	1	2	3	4	5	6	7
1. Adequacy	3.35	0.49							
2. Instruction	3.30	0.79	0.16 [−0.03, 0.33]						
3. Differentiation	2.74	1.06	0.20^*^ [0.02, 0.37]	0.10 [−0.09, 0.28]					
4. Rewards	3.83	0.50	−0.05 [−0.23, 0.13]	0.09 [−0.10, 0.27]	0.00 [−0.18, 0.18]				
5. Motivation	2.18	0.77	0.20^*^ [0.01, 0.37]	−0.00 [−0.18, 0.18]	0.16 [−0.03, 0.33]	−0.14 [−0.32, 0.04]			
6. Evaluation SRL strategy	2.22	0.74	0.13 [−0.05, 0.31]	−0.04 [−0.22, 0.15]	0.13 [−0.05, 0.31]	0.13 [−0.06, 0.30]	0.48^**^ [0.32, 0.61]		
7. Repetition SRL strategy	2.61	0.70	0.12 [−0.07, 0.29]	0.03 [−0.15, 0.22]	0.11 [−0.07, 0.29]	−0.16 [−0.33, 0.03]	0.41^**^ [0.24, 0.55]	0.43^**^ [0.27, 0.57]	
8. Metacognition	1.54	0.63	0.15 [−0.03, 0.33]	0.00 [−0.18, 0.18]	0.29^**^ [0.12, 0.45]	0.01 [−0.17, 0.20]	0.26^**^ [0.08, 0.42]	0.35^**^ [0.18, 0.50]	0.17 [−0.01, 0.34]

*M* and SD are used to represent mean and standard deviation, respectively. Values in square brackets indicate the 95% confidence interval for each correlation. The confidence interval is a plausible range of population correlations that could have caused the sample correlation (Cumming, 2014). ^*^indicates *p* < 0.05. ^**^indicates *p* < 0.01.

In contrast to the primary students’ expressions in the SRL test instrument, the quality characteristics of the TELE ANTON reached overall high medians (Figure 1, left box plot). Hence, the students rated the characteristics of ANTON with averages of *M* = 3.35 for adequacy, *M* = 3.30 for instruction, *M* = 2.74 for differentiation, and *M* = 3.83 for rewards. For the reward system, we found a ceiling effect with a high expression rate for excellent agreement with the TELE. Additionally, Table 3 highlights that for the subscales of the quality characteristics of ANTON, only adequacy and differentiation were significantly correlated, *r* = 0.20 (*p* < 0.05). The lack of correlation between other subscales of the test instruments emphasizes that good separation exists and therefore no confounding between the subscales. Moreover, as a first indicator of how SRL profiles and their rating of ANTON’s characteristics may be related, we also analyzed intercorrelations of the subscales. The results in Table 3 indicate that especially motivation and adequacy are significantly correlated, *r* = 0.20 (*p* < 0.05), as well as metacognition and differentiation, *r* = 0.29 (*p* < 0.01).

**Figure 2:**
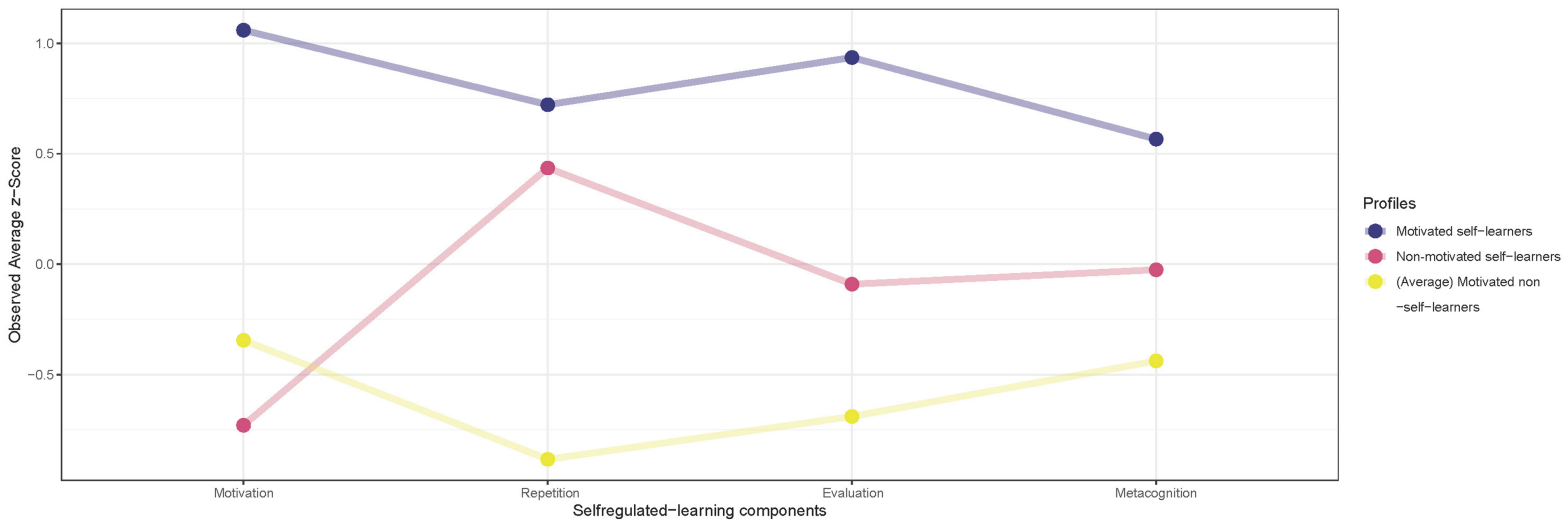
Observed average *z*-score for the three self-regulated learning (SRL) profiles: Motivated self-learners (blue), Non-motivated self-learners (red), and (Average) motivated non-self-learners (yellow).

Based on these findings, we performed a cluster analysis, as described in Section 3.4, to classify students’ SRL profiles to represent groups of primary students who rated their SRL abilities, including their metacognition and motivation to learn mathematics similarly. During this multi-level analysis, we chose a three-cluster solution as the best-fitting model for our data. Table 4 shows the details of the model by displaying the mean scores of the SRL components and highlights that no significant differences exist in the distribution of students among the three-cluster, namely, SRL profiles. Additionally, Figure 2 visualizes the mean *z*-scores for the different subscales of SRL components for the three-profile solution used to entitle and characterize the three primary students’ SRL profiles:

1. The 37 (of 115) students in the first profile were labeled as *motivated self-learners* since they showed the highest self-esteem in their own SRL strategy repetition (0.72), SRL strategy evaluation (0.94), and motivation (1.06), although metacognition was clearly less prominent (0.57).
2. The 32 students in the second profile were labeled as *non-motivated self-learners*, as they rated themselves lowest in motivation (−0.73) but average in their SRL strategy evaluation (−0.09) and metacognition (−0.03) and high in their SRL strategy repetition (0.44), which is close to the motivated self-learners’ expression.
3. The 46 primary schoolchildren in the third profile were labeled as *(average) motivated non-self-learners*, as they rated the SRL strategies repetition (−0.88), evaluation (−0.69), and metacognition (−0.34) as lowest but rated their motivation (−0.34) as slightly higher than the non-motivated self-learners.

**Table 4:** Three-cluster solution with mean scores for the quality characteristics and the specification of absolute and relative (in percentage) numbers of students in the clusters.

Profiles	Mean scores (standard deviation) of the SRL components regarding their profiles				Person (absolute)	Percent in profile (%)
	Repetition	Evaluation	Metacognition	Motivation		
Motivated self-learners	0.72 (0.77)	0.94 (0.81)	0.57 (1.32)	1.06 (0.67)	37	32.17
Non-motivated self-learners	0.44 (0.63)	−0.09 (0.69)	−0.03 (0.74)	−0.73 (0.61)	32	27.83
(Average) motivated non-self-learners	−0.88 (0.65)	−0.69 (0.68)	−0.44 (0.55)	−0.34 (0.70)	46	40.00

### 4.2. Influences of students’ SRL profiles on their rating of the TELE

After the analysis of the student profiles, the second research question addresses the extent to which student profiles differ in the ratings of the quality characteristics of a TELE.

**Figure 3:**
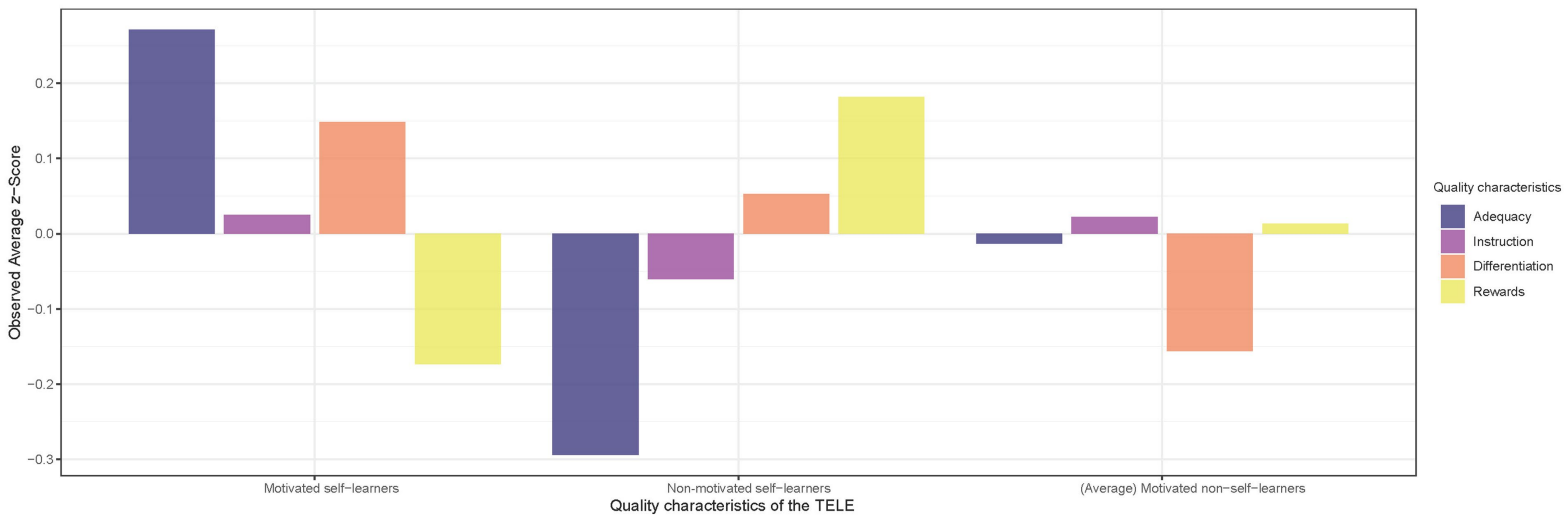
Observed average *z*-score for the rating of the quality characteristics differentiated between the three self-regulated learning (SRL) profiles.

To evaluate the second research question, we focused on the mean *z*-scores of the four quality characters of the TELE ANTON regarding the three primary students’ SRL profiles (Figure 3). On a descriptive level, only small differences in the quality characteristics instruction appear, whereas strong differences occur for the differentiation characteristic. Motivated self-learners rated this TELE characteristic the highest compared to the other student groups and motivated non-self-learners the lowest. Moreover, a difference in the primary schoolchildren’s ratings can be observed for the characteristic rewards. Motivated self-learners rated this characteristic the lowest, whereby non-motivated self-learners rated it the highest. Lastly, the characteristic adequacy was also rated differently by the three SRL profiles. While motivated self-learners rated it highest, non-motivated self-learners rated this characteristic lowest. To test the above observations on how the primary students’ SRL profiles differ in the rating of quality characteristics of ANTON, we performed a Kruskal–Wallis Test. After adjusting the *p*-values with a Bonferroni correction, it became evident that the null hypothesis had to be rejected that the profiles of motivated self-learners and non-motivated self-learners did not differ (*z* = 7.628, *p* = 0.20). Specifically, the Kruskal–Wallis Test was statistically significant for the quality characteristic adequacy (*p* < 0.05), meaning that motivated self-learners and non-motivated self-learners rate adequacy significantly differently.

## 5. Discussion

The study aimed to explore the SRL profiles of primary schoolchildren based on their self-estimated SRL strategies, including metacognition and motivation, and focused on examining differences between these students’ SRL profiles regarding their rating of the quality characteristics of the TELE ANTON. The results for the first research question show that it was possible to identify three student SRL profiles: motivated self-learners, non-motivated self-learners, and the (average) motivated non-self-learners. Therefore, we found two groups of primary schoolchildren who deemed their SRL abilities good but differed regarding their motivation to learn and achieve in mathematics. Altogether, the SRL strategies repetition and evaluation were positively correlated, a finding theoretically assumed by Pintrich and Garcia (1994), Artelt et al. (2001), Boekaerts et al. (2000), and Zimmerman (2000). The motivated self-learners rated all SRL components very high and thus reflected students who probably cope very well in strongly self-regulated learning environments such as TELE. Conversely, the non-motivated self-learners esteemed themselves quite well in the domains of general SRL strategies and metacognition, whereas they appeared less motivated in mathematics. Therefore, primary students with this SRL profile should normally be able to handle self-regulated learning environments such as TELE well but may tend to abandon, delay, or fail mathematical activities in TELEs due to their lack of motivation, which enriches the research of, for example, Garon-Carrier et al. (2016). In this context, it appears particularly relevant to motivate the non-motivated self-learners more strongly when using TELE in mathematics, which will be a contemporary task of mathematics educators (Schrunk et al., 2014; Beal and Rosenblum, 2018). Students belonging to the group of (average) motivated non-self-learners rated themselves lowest in their SRL abilities but medium-high for motivation. In this case, the children’s motivation may help develop their cognitive and metacognitive SRL strategies in mathematics education to encourage them to learn mathematics in self-regulated TELEs.

The results for the second research question indicate that the primary students with the different SRL profiles rated the quality characteristics of the TELE differently. Concerning the mathematical adequacy of the TELE, a statistically significant difference exists between motivated self-learners and non-motivated self-learners. One possible reason for this could be the focus of ANTON, as with many other applications, on a declarative rather than a conceptual or relational understanding of mathematics (Larkin and Milford, 2018). These so-called *drill and practice* tasks in TELE could lead to differences in the rating of ANTON’s adequacy due to the dissimilarity of these two SRL profiles in motivation. While motivated self-learners may tend to acknowledge the adequacy of a TELE because they generally enjoy mathematics, the non-motivated self-learners probably wish for more customization options to meet their needs for mathematical learning processes (Schrunk et al., 2014). Additionally, other differences existed between the students’ ratings of the TELE’s characteristics, which were visible but not statistically significant. For example, differences appeared to exist regarding the characteristic of differentiation between motivated self-learners and motivated non-self-learners. While the motivated self-learners rated ANTON’s differentiation options as high, the motivated non-self-learners evaluated them as quite low, which can be explained by these primary students having a greater need for diverse differentiation methods such as the social, quantitative, media, and qualitative ones presented by Krauthausen (2018) or Tomlinson (2005), which tailor their self-regulated mathematics learning in TELEs (Webb, 2005; Winne and Azevedo, 2014; Walter and Dexel, 2020). Hence, the differentiation ANTON provides is currently insufficient for the group of motivated non-self-learners and must either be technically updated or enhanced by the expertise of mathematics educators. Furthermore, the difference between motivated and non-motivated self-learners in the quality characteristic rewards such as DGBL elements is intriguing, supporting the recent research results of Vankúš (2021), Zhao et al. (2021), Hussein et al. (2022), and Fadda et al. (2022). While motivated self-learners rate the quality of the implemented reward system in ANTON as low (and perhaps have less need for rewards), non-motivated self-learners rated the rewards as high. It can be assumed that non-motivated self-learners, who have lower intrinsic motivation overall, need an extrinsic stimulus from the rewards to learn well in digital learning environments (Pekrun et al., 2002) and manage the mathematical adequacy of the TELE (Bernacki et al., 2011; Faber et al., 2017). The questions raised in this section should be addressed by further research focusing on a larger sample of primary schoolchildren than in this study as well as other frequently used TELEs in mathematics.

On a methodological level, the test statistics of the test instruments were satisfactory for most items. The descriptive statistics results indicate that the newly developed test instruments for assessing the quality characteristics of mathematical TELEs and the adapted test instruments with reduced scales for assessing students’ self-regulatory strategies and metacognitive and motivational skills are mainly suitable for testing primary school students. However, due to reliability issues for two items, we reduced the motivation scale from three to two items and excluded the SRL control strategy scale. This item requires a revision and larger sample sizes of primary schoolchildren learning in TELEs.

In conclusion, our results emphasize that primary students with specific SRL profiles differ in their ratings of the quality characteristics of TELEs, such as adequacy, instructions, differentiation, and rewards. In this study, the highly rated evaluation of ANTON indicates that using TELEs as a supplement to the traditional mathematics classroom (and beyond that in informal educational settings) can contribute to the quality of self-regulated mathematics learning. However, the presented results showed that the students also evaluated this exemplary TELE critically. Here, the fit between the application, in terms of adequacy, differentiation, and rewards, and the student’s SRL abilities and motivation play an important role. Since TELEs such as ANTON often have technical and administrative limitations that are currently not fully (or cannot be) resolved, the options for individualized self-regulated digital learning are limited. Methods to improve a TELE in this way include machine learning methods and intelligent tutoring systems (e.g., Kulik and Fletcher, 2016). However, full individualization of TELEs to meet students’ needs regarding their SRL and motivation is probably impossible. This indicates that educators must evaluate TELEs from a pedagogical perspective to reveal which parts of the broad spectrum of learning opportunities for learning mathematics could be achieved with TELEs and which parts cannot be accomplished *via* this method.

## Data availability statement

The raw data supporting the conclusions of this article will be made available by the authors, without undue reservation.

## Ethics statement

Ethical review and approval was not required for the study on human participants in accordance with the local legislation and institutional requirements. Written informed consent to participate in this study was provided by the participants’ legal guardian/next of kin.

## Author contributions

All authors listed have made a substantial, direct, and intellectual contribution to the work and approved it for publication.

## Conflict of interest

The authors declare that the research was conducted in the absence of any commercial or financial relationships that could be construed as a potential conflict of interest.

## Publisher’s note

All claims expressed in this article are solely those of the authors and do not necessarily represent those of their affiliated organizations, or those of the publisher, the editors and the reviewers. Any product that may be evaluated in this article, or claim that may be made by its manufacturer, is not guaranteed or endorsed by the publisher.
